# Reward-Related Behavioral Paradigms for Addiction Research in the Mouse: Performance of Common Inbred Strains

**DOI:** 10.1371/journal.pone.0015536

**Published:** 2011-01-10

**Authors:** Lauren Lederle, Susanna Weber, Tara Wright, Michael Feyder, Jonathan L. Brigman, Hans S. Crombag, Lisa M. Saksida, Timothy J. Bussey, Andrew Holmes

**Affiliations:** 1 Section on Behavioral Science and Genetics, Laboratory for Integrative Neuroscience, National Institute on Alcohol Abuse and Alcoholism, National Institutes of Health, Rockville, Maryland, United States of America; 2 Behavioural and Clinical Neuroscience Research Group, School of Psychology, University of Sussex, Brighton, United Kingdom; 3 Department of Experimental Psychology and the Medical Research Council and Wellcome Trust Behavioral and Clinical Neuroscience Institute, University of Cambridge, Cambridge, United Kingdom; University of Chicago, United States of America

## Abstract

The mouse has emerged as a uniquely valuable species for studying the molecular and genetic basis of complex behaviors and modeling neuropsychiatric disease states. While valid and reliable preclinical assays for reward-related behaviors are critical to understanding addiction-related processes, and various behavioral procedures have been developed and characterized in rats and primates, there have been relatively few studies using operant-based addiction-relevant behavioral paradigms in the mouse. Here we describe the performance of the C57BL/6J inbred mouse strain on three major reward-related paradigms, and replicate the same procedures in two other commonly used inbred strains (DBA/2J, BALB/cJ). We examined Pavlovian-instrumental transfer (PIT) by measuring the ability of an auditory cue associated with food reward to promote an instrumental (lever press) response. In a separate experiment, we assessed the acquisition and extinction of a simple stimulus-reward instrumental behavior on a touchscreen-based task. Reinstatement of this behavior was then examined following either continuous exposure to cues (conditioned reinforcers, CRs) associated with reward, brief reward and CR exposure, or brief reward exposure followed by continuous CR exposure. The third paradigm examined sensitivity of an instrumental (lever press) response to devaluation of food reward (a probe for outcome insensitive, habitual behavior) by repeated pairing with malaise. Results showed that C57BL/6J mice displayed robust PIT, as well as clear extinction and reinstatement, but were insensitive to reinforcer devaluation. DBA/2J mice showed good PIT and (rewarded) reinstatement, but were slow to extinguish and did not show reinforcer devaluation or significant CR-reinstatement. BALB/cJ mice also displayed good PIT, extinction and reinstatement, and retained instrumental responding following devaluation, but, unlike the other strains, demonstrated reduced Pavlovian approach behavior (food magazine head entries). Overall, these assays provide robust paradigms for future studies using the mouse to elucidate the neural, molecular and genetic factors underpinning reward-related behaviors relevant to addiction research.

## Introduction

The availability of valid and reliable methods for studying incentive learning and other reward-related behaviors in experimental animals is essential to furthering our understanding of the neural, genetic and molecular basis of addiction. To this end, various rodent behavioral assays have been developed to probe core processes that underlie the natural motivation to seek reward, and that are theorized to go awry in drug abuse and addiction [1], [2].

Amongst the rodent paradigms relevant to addiction are those which measure Pavlovian-instrumental transfer (PIT) – a process by which, through their association with reward, previously neutral stimuli can instigate or energize an existing instrumental reward-seeking response [3]. PIT can be observed in humans [4] as well as various laboratory animal species and is mediated by analogous corticolimbic circuitry (prefrontal cortex (PFC), ventral striatum, amygdala) that become functionally aberrant in drug addicts [5]. The mechanisms subserving PIT likely overlap with those that drive craving and relapse in response to drug-related cues and paraphernalia in addicts [6], [7].

Maintenance and relapse in drug addiction is most often modeled in rodents using extinction and reinstatement procedures. Extinction occurs when the frequency of an instrumental response is reduced by removing the previous response-contingent reward. The extinguished response can be subsequently reinstated by various events, including stressors, presentation of reward-associated cues (‘conditioned reinforcers’) or brief exposure to the reward (‘priming’) [8]. Similar to PIT, both extinction and reinstatement are mediated by the corticolimbic circuits that are persistently altered by drug exposure and implicated in addiction [9], [10].

More recently, and spurred by theoretical developments, there has been a growing interest in understanding the role of habit learning and behavioral flexibility in compulsive drug use and addiction [5], [11]. An operational definition of habit is behavior that is insensitive to changes in outcome value, as measured by, for example, the maintenance of behavioral responding after the reward that maintained it has been devalued (e.g., by selective satiation or pairing with illness) [12]. Like PIT and reinstatement, the neural circuitry underlying the formation and expression of habitual behaviors has, to some extent, been mapped and includes subregions of the PFC and dorsal striatum [13], [14]. However, the molecular and genetic bases, not just of habitual behavior, but of other reward-related behaviors including PIT, extinction and reinstatement, are currently less well understood. Elucidating these factors is a key goal for understanding the pathophysiology of addiction and identifying potential therapeutic targets.

In this context, the mouse is a uniquely informative model species for elucidating the molecular and genetic basis of motivated behavior, addictions and other neuropsychiatric disease states [15]–[17]. To date, however, there have been relatively few studies of the aforementioned behaviors using mouse models, in part, because the methodology and procedures for doing so are poorly characterized. Therefore, the aim of the present study was to assess the utility of various operant-based methodologies and procedures to characterize reward-related behavior in mice, focusing on the three aforementioned paradigms: auditory cue-induced PIT; acquisition, extinction and reinstatement of a stimulus-response (S–R) instrumental response; and malaise-induced reinforcer devaluation. To broadly assess the generalizability of our behavioral methods and procedures, we replicated the same procedures in three different inbred mouse strains commonly used in behavioral genetics and/or drug research (C57BL/6J, DBA/2J, BALB/cJ). Our findings show that, with few exceptions, these procedures provide robust assays for the study of complex aspects of motivated behavior in mice relevant to understanding addiction.

## Materials and Methods

### Subjects

Subjects were male C57BL/6J, DBA/2J and BALB/cJ mice obtained from The Jackson Laboratory (Bar Harbor, ME). These strains of inbred mice were selected on the basis of 1) their frequent use in behavioral neuroscience as the genetic backgrounds for mouse mutant lines, 2) their inclusion as “group A” priority strains in the Mouse Phenome Project - an international effort to provide the biomedical research community with phenotypic data on the most commonly used mouse strains (www.jax.org/phenome), and 3) our previous characterization of these strains for relevant behavioral phenotypes, including ethanol-sensitivity and consumption [18], [19], sensorimotor gating [20] and various emotion-related processes [21]–[23].

Mice were aged 8–9 wks at the start of the experiments and housed in pairs in a temperature- (72±5°F) and humidity- (45±15%) controlled *vivarium* under a 12∶12 hr light/dark cycle (lights on 0600 h). The number of mice used in each experiment is indicated in the relevant figure legends. For all experiments, mice were slowly reduced in weight (over approximately 1 week) prior to testing and then maintained at 85% of their free-feeding body weight through completion of testing. All experimental procedures were approved by the National Institute on Alcohol Abuse and Alcoholism Animal Care and Use Committee under animal study protocol #LIN-AH-21 and followed the National Institutes of Health guidelines outlined in ‘Using Animals in Intramural Research’ and the local Animal Care and Use Committees.

### Pavlovian-instrumental transfer

Testing was conducted in 21.6×17.8×12.7 cm operant chambers (model #ENV-307W, Med Associates, St. Albans, VT) housed within sound and light attenuating enclosures (Med Associates model #ENV-022MD). The grid floor of the chamber was covered with solid Plexiglas to facilitate ambulation. A pellet dispenser delivering a 14-mg reward pellet (catalogue #F05684; BioServ) into a food magazine was located at one end of the chamber. An infrared photo-beam was located inside the receptacle to detect head entries (HEs) into the magazine. Ultra-sensitive response levers (model # ENV-310W) were located ∼5 cm to each side of the magazine. Speakers emitting either a ∼85 dB broadband white-noise cue (Med Associates model # ENV-325SW) or a 3 kHz pure tone cue (Med Associates model # ENV-324W) were positioned ∼5 cm above the levers. MED-PC software (Med Associates) controlled cue presentation and reward delivery and recorded HEs and lever presses.

**Figure 1:**
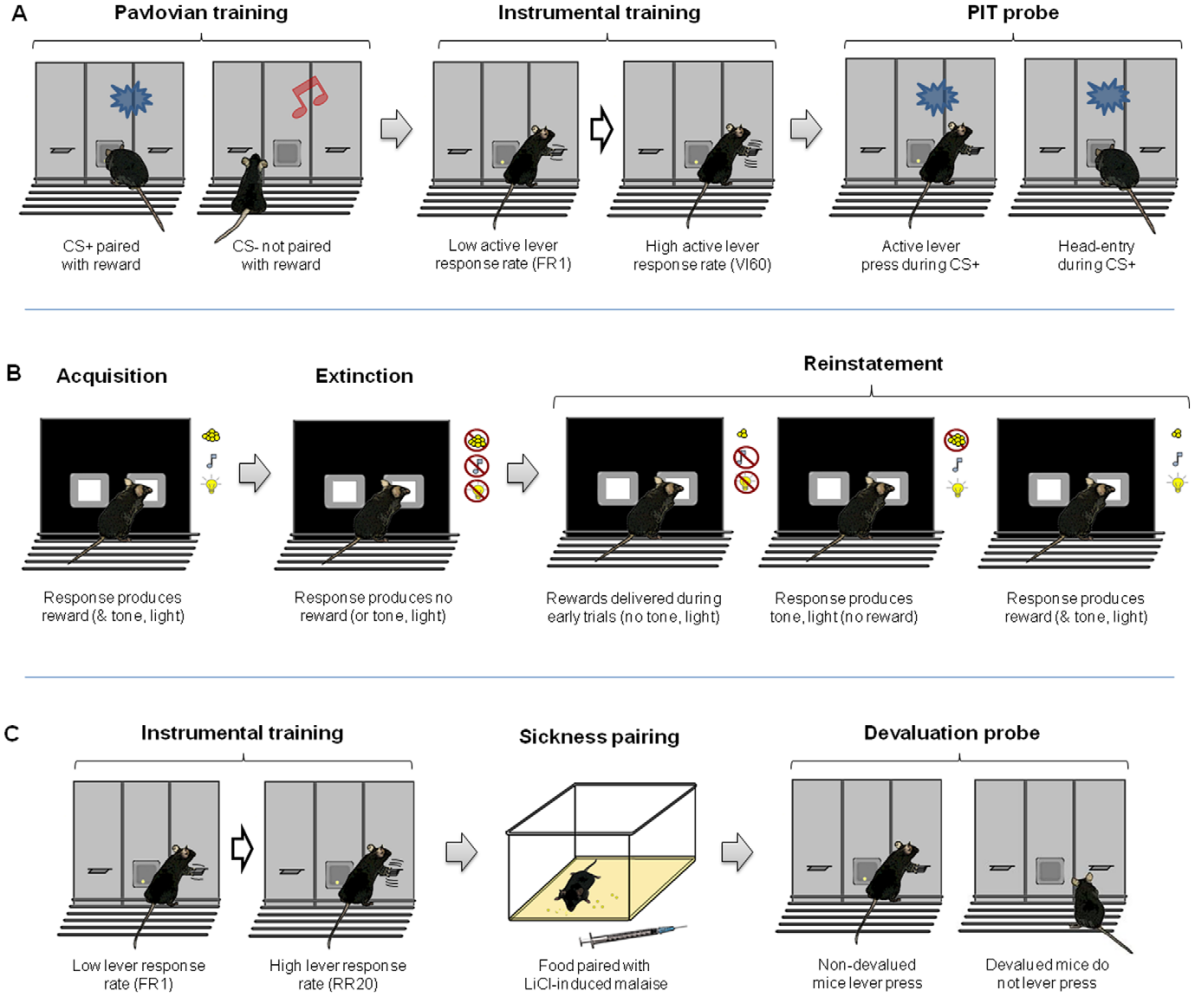
Cartoon depictions of the main procedural elements of the reward-related paradigms studied. (**A**) Pavlovian-instrumental transfer. Mice were first trained to discriminate between one auditory cue (CS+) which was predictive of the delivery of a food reward and a second auditory cue (CS−) not associated with reward. Instrumental training, starting with a continuous schedule of reinforcement, progressed through increasingly sparse variable interval schedules of reinforcement to increase the rate of pressing on the active lever (AL) delivering food reward. Presses on a second, inactive (control) lever (IL) had no programmed consequences. During the PIT probe test, lever presses during presentations of the CS+, CS− and inter-trial interval (ITI) periods were recorded as well as Pavlovian approach behavior (head-entries (HE) into the magazine). (**B**) Acquisition, extinction and reinstatement of an instrumental response. Mice were trained to respond to a visual stimulus on a touchscreen to obtain a food reward (delivery of which was concomitant with a compound light/tone stimulus to serve as a conditioned reinforcer). During extinction training, responses produced no reward or conditioned reinforcement. Response were subsequently reinstated either by non-contingent delivery of reward and conditioned reinforcers (CR) during the first six of thirty trials (Reward^6^+CR^6^), delivery of the CRs during all thirty trials (CR^30^), or a combination of these two procedures (Reward^6^+CR^30^). (**C**) Malaise-induced reinforcer devaluation. During instrumental training, mice were trained to lever press on a random ratio reinforcement schedule for food reward. Next, on separate session conducted in the home cage, free availability of food pellet was paired with LiCl-induced malaise (Devalued group) or Food and LiCl injections were given unpaired (Non-devalued group). The effect of US-devaluation on lever pressing and magazine head entry was examined in the absence of the primary food reward (i.e. extinction) in the conditioning chambers.

Testing began with a single 30 min session to habituate mice to the chamber and to the intermittent availability (on a random interval (RI) 60 sec schedule) of food pellets (unconditioned stimulus or US) in the recessed magazine (levers were unavailable). Mice then underwent daily Pavlovian discrimination training sessions to associate one auditory cue (conditioned stimulus, CS+) with the delivery of the US, and a second cue (CS−) with the absence of reward (Figure **1A**, left). For half of the subjects, the CS+ was the white-noise and the CS− was the pure tone, and vice versa for the other half. Sessions were 41 min in duration and 1 session was conducted each day. Each session consisted of 5× (120-sec) CS+ and 5× (120-sec) CS− trials, occurring in random order, and each trial was separated by an inter-trial interval (ITI) lasting, on average, 120 sec (variable interval (VI120)). Within each CS+ presentation, 4 USs were delivered on average every 30 sec (RI30) and no USs were delivered during the CS− presentations. Levers remained unavailable. HEs were measured for each session until the subject met the dual criterion for acquiring the Pavlovian discrimination of: 1) >200 HEs/session into the receptacle, and 2) of HEs made during the first 15 sec of CS presentations (i.e., when no food was presented), >85% were made during CS+ presentations.

Next, mice were trained to press one of 2 levers to receive response-contingent delivery of the US (Figure **1A**, center). Responses on the reinforced lever (designated the ‘active lever,’ AL) produced the US, while responses on the non-reinforced, control lever (designated the ‘inactive lever,’ IL) had no programmed consequences. Left vs. right designation of the AL was counterbalanced across subjects. Sessions were conducted daily and lasted 30 or 60 min and the number of AL and IL presses was measured for the full session. Instrumental training progressed along 4 increasingly sparse schedules of reinforcement designed to produce stable and high rates of AL responding using the following steps: i) Fixed ratio 1 (FR1) schedule in which every AL press was rewarded with a single US and the criterion for progression to the more sparse schedule was >20 AL responses in a 60-min session; ii) Variable interval (VI15) schedule, in which each AL press was rewarded on average every 15 sec, and the criterion for progression to the more sparse schedule was >150 AL responses in a 30-min session; iii) Variable interval (VI30) schedule, in which each AL press was rewarded on average every 30 sec, and the criterion for progression to the more sparse schedule was >300 AL responses in a 30-min session; iv) Variable interval (VI60) schedule, in which each AL press was rewarded on average every 60 sec, and the criterion for completion of the instrumental training phase for PIT was 1) making >400 AL responses in a 30-min session, and 2) <20% response variability across 3 consecutive VI60 sessions.

After instrumental training was completed, a Pavlovian-instrumental transfer (PIT) probe test (Figure **1A**, right) was conducted under extinction conditions such that AL and IL responses had no programmed consequences. The probe test was 46 min in duration, beginning with a 6-min no-CS period followed by 5× (120-sec) CS+ and 5× (120-sec) CS− presentations, each separated by a 120-sec inter-trial-interval (no US delivered). CS+ and CS− trials were alternated, with CS+ trials occurring first for half of the subjects. Responses were measured for CS+, CS− trial and ITI periods separately to allow for the number of AL presses, IL presses and HEs to be compared across the CS+ and CS− presentations and ITI period using repeated measures analysis of variance (ANOVA) followed by Fishers LSD *post hoc* tests. In addition, the number of AL versus IL presses during the CS+, CS− and ITI was compared by paired Student's t-tests.

### Acquisition, extinction and reinstatement of an instrumental response

This behavioral assay had 3 components: 1) acquisition of a simple instrumental S-R association reinforced by food (US) reward, 2) extinction of instrumental responding by removal of reinforcement, and 3) assessment of reinstatement of instrumental responding in the presence of the primary (i.e., food) reinforcer, the food-associated light and tone cues (conditioned reinforcers), or a combination of the primary + conditioned reinforcer. The acquisition and extinction procedures have been described previously [23]–[25].

The apparatus was the same as for the PIT assay except that instead of levers, the response device consisted of a touch-sensitive LCD screen located at the opposite end of the operant chamber from the food magazine. The touchscreen (Light Industrial Metal Cased TFT LCD Monitor, Craft Data Limited, Chesham, U.K.) was covered by a opaque Plexiglas panel with 2×5 cm^2^ ‘cut-outs’ 6.5 cm above the chamber floor that outlined 2 discrete stimulus presentation windows separated by 0.5 cm (Figure **1B**, left). Presentation of visual stimuli on the touchscreen and recording of touches was controlled by custom software (‘MouseCat’, L.M. Saksida) as previously described [25]–[27].

Daily testing began with a single 30 min habituation session to the chamber and intermittent delivery of food pellets into the magazine. This was followed by a 3-phase pre-training procedure to shape the instrumental response. During phase 1, visual stimuli (shape randomly varied) were presented pseudorandomly in 1 of the touchscreen windows for 10 sec, on average every 15 sec, immediately followed by delivery of a single US. Reward delivery was concomitant with the compound presentation of 2 cues, consisting of a 2-sec 65 dB auditory tone and illumination of the food magazine, that were designed to serve as explicit secondary or conditioned reinforcers during the tests for reinstatement (see below). Food reward retrieval was detected by the first HE following delivery, and this also initiated the next trial. In phase 2, delivery of the food US was made contingent on the mouse making physical contact with the touch-sensitive LCD screen in the window (pseudorandomly determined) displaying the randomly-shaped visual stimulus (presented pseudorandomly in 1 of the touchscreen windows) and the response also initiated the subsequent trial. Phase 3 was the same as phase 2 with the additional requirement that trial initiation (after the first) was dependent upon the mouse making an additional HE into the magazine after reward retrieval and, to discourage indiscriminate responding, the inclusion of a 5 sec lights-out, time-out period after responses into a blank window. To progress through each of the 3 phases, mice were required to retrieve 30 pellets within a 30-min session period.

For the acquisition task proper, mice were required to initiate and respond to either 1 of 2 stimuli (1×2.8 cm^2^ white square per window) over 30 trials (5 sec ITI). Stimuli remained on the screen until a response was made. A response produced a single reward and the CRs. Acquisition criterion was making 30 responses within 12.5 min on each of 5 consecutive sessions.

The assessment of extinction began the session after acquisition criterion was met by monitoring (previously food reinforced) instrumental responding in the absence of food reinforcement. The visual touchscreen panel stimuli were presented over 30 trials and remained on-screen for 9 sec or until a response was made. A response produced no food US reinforcement or the explicit conditioned reinforcers (Figure **1B**, center). Extinction sessions continued until mice met a criterion of ≥77% omissions (non-responses) per session on 2 consecutive sessions.

The session after attaining extinction criterion, mice were assessed for reinstatement of instrumental responding. Separate groups of mice were tested on 1 of 3 reinstatement procedures (Figure **1B**, right): 1) Reinstatement by re-exposure to the US was tested by delivering a US (and the conditioned reinforcers) on *only* the first 6 trials of a 30-trial session after a response was made or, in the absence of a response, following the offset of the stimulus after 9 sec (Reward^6^+CR^6^ condition). During the remaining 24 trials no food or cues were presented. 2) Reinstatement by re-introduction of the conditioned reinforcers *alone* after a response or, in the absence of a response following the offset of the stimulus after 9 sec, *on all 30 trials* (CR^30^ condition). 3) Reinstatement by 6 x US reinforced priming trials at the start of the session (as in 1 above) and presentation of the conditioned reinforcers on all 30 trials (as in 2 above) (Reward^6^+CR^30^ condition). Reinstatement of instrumental responding was determined by calculating the difference in responding during the reinstatement session and responding during the final day of extinction, and analyzed using paired Student's t-tests.

### Reinforcer devaluation

For assaying habit-learning using the devaluation procedure, the apparatus was the same as that used to test for PIT. Procedures were based upon those previously used to test for reinforcer devaluation in rats [28], [29] and C57BL/6J mice [30]. Daily testing began with 2 x habituation sessions to the chamber and intermittent delivery of the food US into the magazine on a RI30 sec schedule (levers unavailable). Mice were then trained on a discriminated instrumental task to press a lever to obtain the US (Figure **1C**, left) using the same instrumental training protocol as previously employed to test satiety-induced reinforcer devaluation in C57BL/6J mice [30]. Left versus right designation of the available lever was counterbalanced across mice. Training began on a continuous, FR1 reinforcement schedule and mice were required to make 5, 15 and finally 30 responses on 3 consecutive sessions (1 session/day). Sessions ended when mice completed the required number of responses or 90 min elapsed. All mice then received 1×30 min session on a random ratio schedule in which responses were reinforced on average every 10 lever presses (RR-10), followed by 3×30-min sessions on a RR-20 schedule. Because the degree of exposure to the reinforcer rather than the number of responses made influences devaluation [see 28], the maximum number of reinforcers that could be earned per session during RR training was limited to 30. Sessions ended when subjects earned 30 reinforcers or 90 min had elapsed. Next, mice were assigned to a devalued or non-devalued group by matching the number of reinforcers earned during instrumental training.

Beginning the day after completing instrumental training, the US was devalued by repeatedly pairing it with sickness (Figure **1C**, center). Mice assigned to the devalued group were provided with 50 pellets placed in a small cap in their home cage (mice were acclimated to the cap in the home cage 1 day earlier), and after 15 min the cap was removed and the number of pellets consumed recorded. Mice were then injected with 0.15 M LiCl [23] (intraperitoneally in a volume of 20 mL/kg body weight) and placed back into their home cage. Mice assigned to the non-devalued group were injected with LiCl but did not receive pellets. The following day, the non-devalued, but not devalued, group received pellets but neither group was injected with LiCl. These procedures were repeated over the next 2 days and served to equate both exposure to LiCl injection and the availability of pellets between the groups, while explicitly pairing the food and LiCl-induced malaise in the devalued group only.

The day after the 4 day devaluation phase, mice were probed for the effects of devaluation on instrumental responding by measuring the number of lever presses and HEs over a 5-min test session conducted in the absence of food reinforcement, i.e. under extinction conditions (Figure **1C**, right). To verify that US devaluation in the home cage generalized to the operant chamber, consumption of 50 pellets made freely available in the chamber's recessed magazine was measured 6 hr after the devaluation probe. Next to assess the persistence of US devaluation (long-term retention), consumption of 50 pellets provided in the home cage was measured. Consumption, lever and head entry responding for the devalued and non-devalued groups were compared using Student's t-tests.

## Results

### Profile of C57BL/6J mice

#### PIT

**Figure 2:**
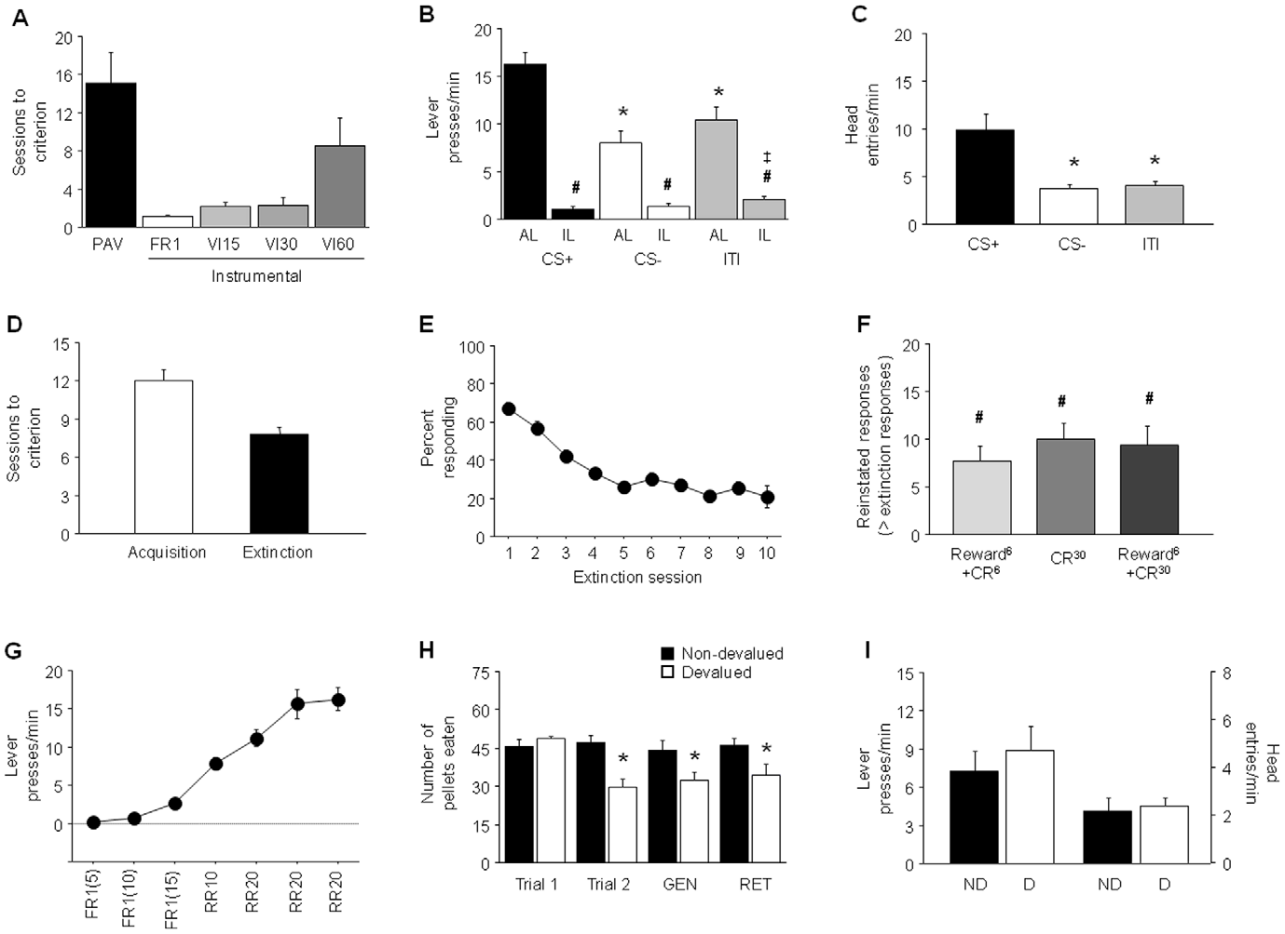
Reward-related behaviors in the C57BL/6J inbred mouse strain. (**A**) Sessions to reach criterion on Pavlovian (PAV) and various schedules of instrumental training prior to the PIT probe (n = 13). (**B**) During the PIT probe, there were more active lever (AL) presses during the CS+ than the CS− trials, or the ITI periods (*p<.05), and more AL presses than inactive lever (IL) presses during the CS+ and CS− trials, and ITI periods (#*p*<.05). There were also more IL presses during the ITI periods than the CS+ (‡*p*<.05). (**C**) More head entries were made during the CS+ than CS− trials, or ITI periods (*p<.05) (n = 12). (**D**) Training sessions to acquire and extinguish a simple stimulus-reward instrumental response (n = 31). (**E**) Percent responding across extinction sessions. (**F**) Reinstatement of operant behavior by the Reward^6^+CR^30^, CR^30^ and Reward^6^+CR^30^ protocols (#*p*<.05 versus responding on last extinction session) (n = 10/condition). (**G**) Lever press responding across instrumental training session prior to devaluation (n = 17). (**H**) Pellets eaten prior to trial 1 and 2 of US-LiCl paired (Devalued) or unpaired (Non-devalued group) session in the home cage, during a context generalization test (GEN) in the conditioning chamber and the home cage retention test (RET) (n = 7–10/group). (**I**) Lever presses and head-entry responses into the magazine did not differ between devalued (D) and non-devalued (ND) groups. FR = fixed ratio, VI = variable interval, RR = random ratio. Data are Means ±SEM.

C57BL/6J mice took on average ∼15 sessions to reach criterion for Pavlovian discriminated approach, although ∼20% of the original sample failed to attain criterion even with extensive training (>45 sessions) and were excluded from further testing. C57BL/6J mice passed through instrumental training in another ∼15 sessions, with the VI60 schedule accounting for over half of the training sessions (Figure **2A**). On the PIT probe test, C57BL/6J mice made significantly more AL presses during presentation of the CS+ than during presentation of the CS− or during the ITI periods (main ANOVA effect of stimulus: F2,24 = 21.17, *p*<.01, followed by *post hoc* tests) (Figure **2B**). In addition, there was significantly more AL pressing than IL pressing during the CS+ (t = 12.39, df = 12, *p*<.01), CS− (t = 5.99, df = 12, *p*<.01) and ITI (t = 5.90, df = 12, *p*<.01). IL pressing was also significantly greater during the ITI than CS+ presentation (main effect of stimulus: F2,24 = 6.85, *p*<.01, followed by *post hocs*). Finally, C57BL/6J mice made significantly more HEs during presentation of the CS+ than either the CS− or ITI (main effect: F2,24 = 15.85, *p*<.01, followed by *post hoc* tests), and the latter did not differ significantly (Figure **2C**).

#### Acquisition, extinction and reinstatement

C57BL/6J mice rapidly acquired the simple stimulus-controlled instrumental responding in ∼12 sessions (Figure **2D**) and, when the reinforcer was subsequently removed, responding rapidly extinguished to criterion (see Methods) within ∼8 sessions (Figure **2E**). All 3 reinstatement procedures produced significant and similar increases in responding relative to the last extinction session: Reward^6^+CR^6^ (t = 4.79, df = 9, *p*<.01), CR^30^ (t = 6.00, df = 9, *p*<.01), and Reward^6^+CR^30^ (t = 4.73, df = 9, *p*<.01) (Figure **2F**); C57BL/6J mice made on average ∼7–10 more responses (out of a possible 30) during reinstatement than extinction.

#### Reinforcer devaluation

C57BL/6J mice showed increasing rates of lever pressing from 1–2 presses/min on the FR schedule, to ∼8 and ∼15 lever presses by the end of RR10 and RR20 training, respectively (Figure **2G**). Mice assigned to both the devalued and non-devalued groups ate most of the 50 pellets offered in their home cage prior to the first devaluation pairing. On the second devaluation pairing, the devalued group consumed significantly fewer pellets than the non-devalued group (t = 3.96, df = 15, *p*<.01) indicating that consumption of the food-pellets had been associated with LiCl-induced malaise experience (Figure **2H**). Nonetheless, during the subsequent devaluation probe in the conditioning chambers no significant differences were found between the devalued and non-devalued groups in the number of lever presses or HEs (Figure **2I**). Importantly, when we subsequently assessed generalization and retention of the food-LiCl pairing association by measuring pellet consumption in the chamber and home cage, respectively, the devalued group consumed fewer pellets compared to the non-devalued group (conditioning chamber, t = 2.45, df = 15, *p*<.01; home cage retention, t = 2.19, df = 15, *p*<.05), again confirming that food-pellets were associated with LiCl-induced malaise in the devalued group (Figure **2H**).

### Profile of DBA/2J mice

#### PIT

**Figure 3:**
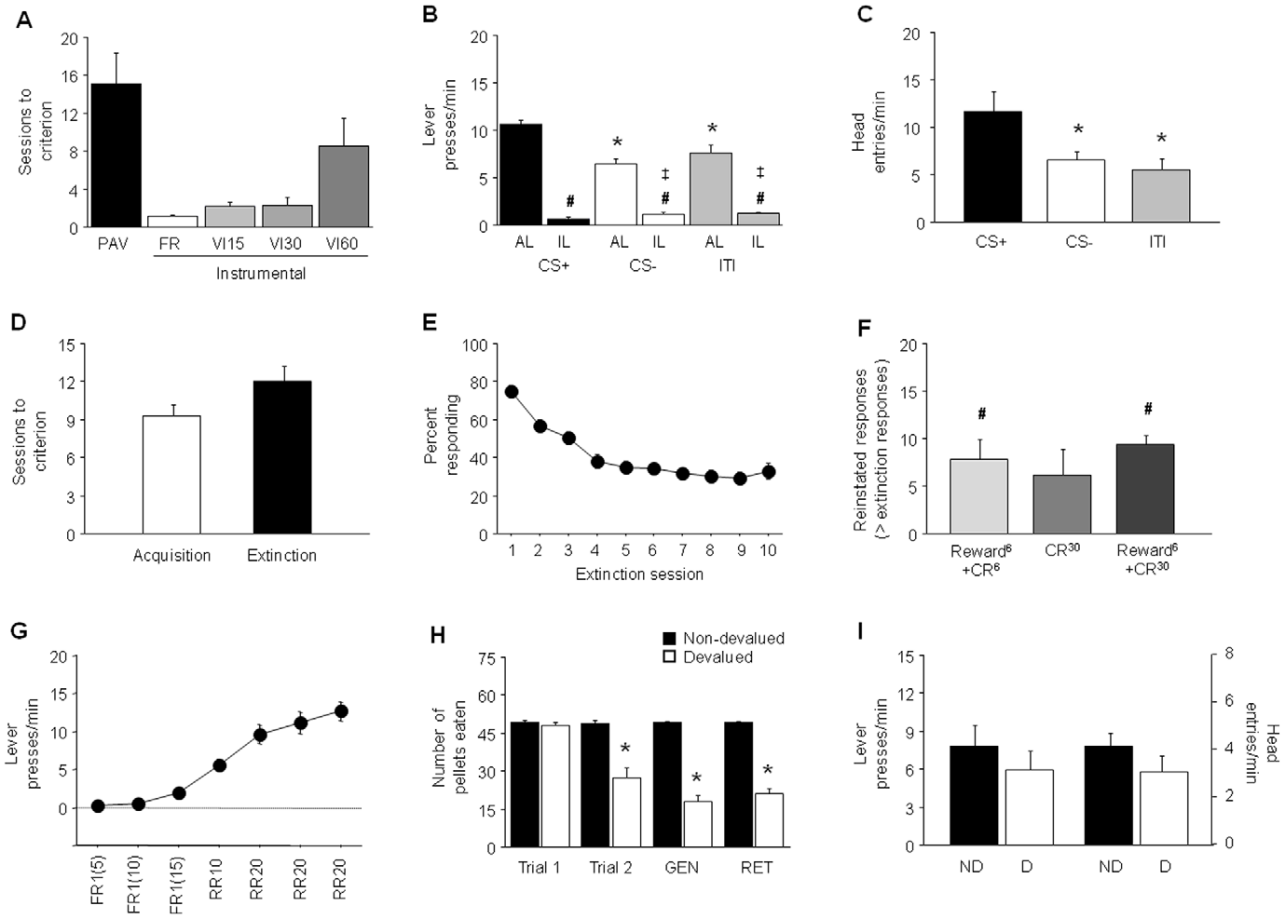
Reward-related behaviors in the DBA/2J inbred mouse strain. (**A**) Sessions to reach criterion on Pavlovian (PAV) and various schedules of instrumental training prior to the PIT probe (n = 12). (**B**) During the PIT probe, there were more active lever (AL) presses during the CS+ than the CS− or ITI (*p<.05), and more AL presses than inactive lever (IL) presses during the CS+, CS− and ITI (#*p*<.05). There were also more IL presses during the ITI than the CS+ and CS− (‡*p*<.05). (**C**) More head entries were made during the CS+ than CS− or ITI (*p<.05) (n = 12). (**D**) Sessions to acquire and extinguish a simple stimulus-reward operant behavior (n = 21). (**E**) Percent responding across extinction sessions. (**F**) Reinstatement of operant behavior by Reward^6^+CR^6^ and Reward^6^+CR^30^ but not CR^6^ protocols (#*p*<.05 versus responding on last extinction session) (n = 6–9/condition). (**G**) Increasing lever pressing with instrumental training prior to devaluation (n = 18). (**H**) Pellets eaten prior to trial 1 and 2 of US-sickness pairing, during a context generalization tests (GEN) and a long-term retention test (RET) (n = 9/group). (**I**) Lever presses and head entries did not differ between devalued (D) and non-devalued (ND) groups. FR = fixed ratio, VI = variable interval, RR = random ratio. Data are Means ±SEM.

While ∼8% of DBA/2J mice failed to reach criterion for Pavlovian discriminated approach even with extensive training, the majority of mice of this strain took ∼15 sessions to show robust Pavlovian discrimination (not shown). A similar number of sessions were needed to complete the instrumental training, largely accounted for by instrumental sessions on the VI60 schedule of reinforcement (Figure **3A**). DBA/2J mice made significantly more AL presses on the PIT probe during CS+ than during CS− trials or during the ITI periods (main effect or stimulus: F2,22 = 14.61, *p*<.01, followed by *post hoc* tests) (Figure **3B**). There was also significantly more AL than IL pressing during the CS+ (t = 18.91, df = 11, *p*<.01), CS− (t = 10.19, df = 11, *p*<.01) and ITI (t = 7.68, df = 11, *p*<.01). DBA/2J mice pressed the IL significantly more during the CS− trials and ITI periods than during CS+ trials (main effect stimulus: F2,22 = 5.95, *p*<.05, followed by *post hocs*). Finally, these mice made significantly more HEs during CS+ trials compared to CS− trials or ITI periods (main effect: F2,22 = 8.81, *p*<.01 followed by *post hoc* tests) (Figure **3C**).

#### Acquisition, extinction and reinstatement

DBA/2J mice rapidly acquired and reached the instrumental response criterion in ∼9 sessions (Figure **3D**) and, extinguished subsequent non-reinforced responding to criterion levels in ∼12 sessions (Figure **3E**). Responding significantly increased during the tests for reinstatement compared to extinction, for the Reward^6^+CR^6^ (t = 7.83, df = 5, *p*<.01) and Reward^6^+CR^30^ (t = 10.37, df = 8, *p*<.01), but responding was not significantly reinstated in the CR^30^ condition (Figure **3F**). DBA/2J made on average ∼7–9 more responses during the Reward^6^+CR^6^ and Reward^6^+CR^30^ reinstatement test compared to extinction levels of responding.

#### Reinforcer devaluation

Lever pressing gradually increased, most clearly during the RR schedules, over instrumental training to ∼12 presses/min (Figure **3G**). Devalued and non-devalued groups both ate most of the 50 pellets prior the LiCl-devaluation procedure and, as expected, the devalued mice consumed significantly fewer pellets than the non-devalued group on the second pairing day (t = 5.50, df = 16, *p*<.01) (Figure **3H**). However, the number of lever presses and HEs did not differ between the devalued and non-devalued groups during the devaluation probe conducted in the conditioning chambers (Figure **3I**). Nonetheless, we confirmed that the LiCl devaluation experience successfully generalized to consumption in the operant chamber (t = 13.71, df = 16, *p*<.01) and the devaluation effect was maintained on the final retention test for consumption back in the home cage (t = 12.74, df = 16, *p*<.01) (Figure **3H**).

### Profile of BALB/cJ mice

#### PIT

**Figure 4:**
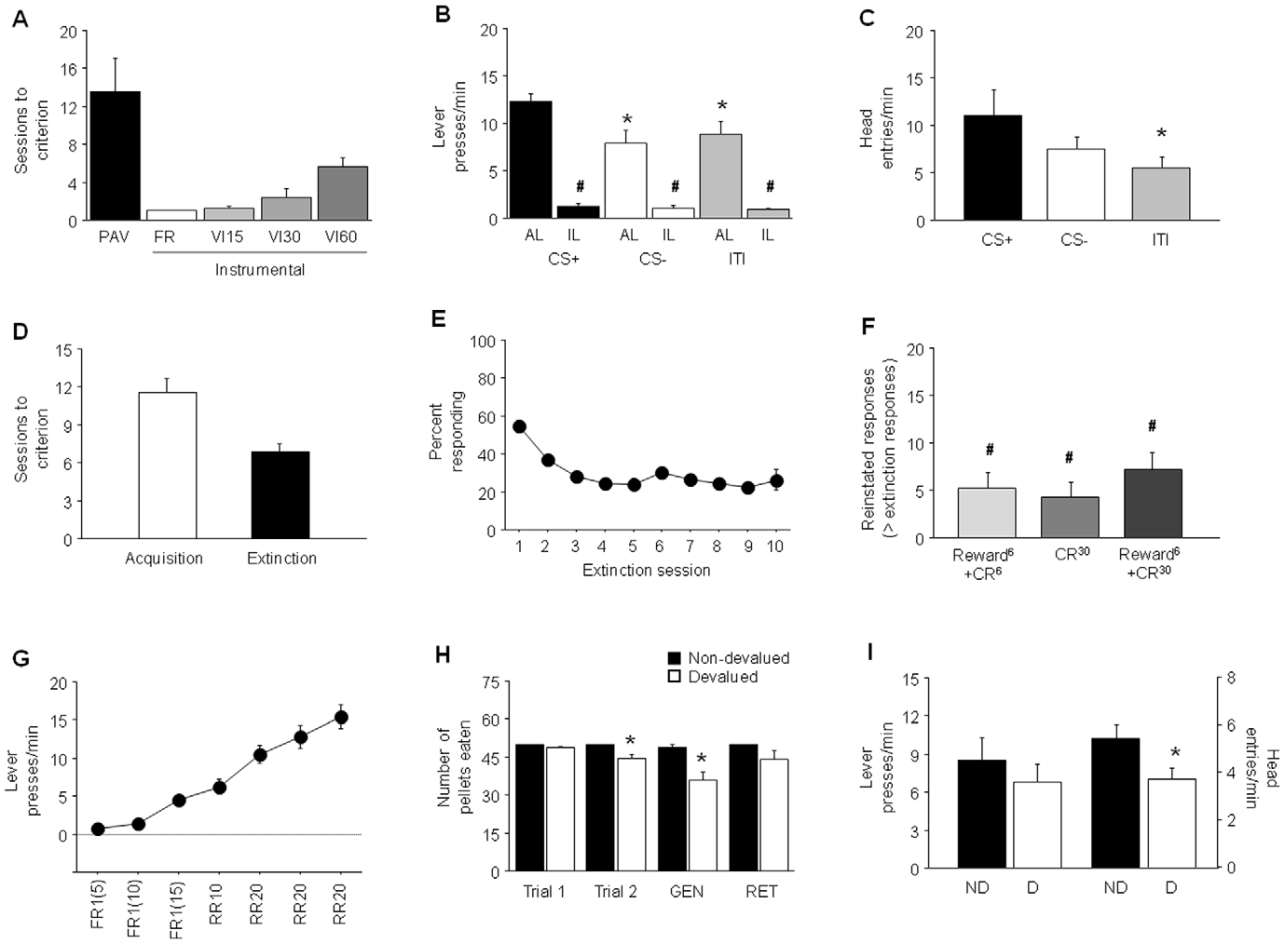
Reward-related behaviors in the BALB/cJ inbred mouse strain. (**A**) Sessions to reach criterion on Pavlovian (PAV) and various schedules of instrumental training prior to the PIT probe (n = 12). (**B**) During the PIT probe, there were more active lever (AL) presses during the CS+ than the CS− or ITI (*p<.05), and more AL presses than inactive lever (IL) presses during the CS+, CS− and ITI (#*p*<.05). There were also more IL presses during the ITI than the CS+ and CS− (‡*p*<.05). (**C**) More head entries were made during the CS+ than CS− or ITI (*p<.05) (n = 12). (**D**) Sessions to acquire and extinguish a simple stimulus-reward operant behavior (n = 28). (**E**) Percent responding across extinction sessions. (**F**) Reinstatement of operant behavior by Reward^6^+CR^6^, CR^6^ and Reward^6^+CR^30^ (#*p*<.05 versus responding on last extinction session) (n = 9–10/condition). (**G**) Increasing lever pressing with instrumental training prior to devaluation (n = 16). (**H**) Pellets eaten prior to trial 1 and 2 of US-sickness pairing, during a context generalization tests (GEN) and a long-term retention test (RET) (n = 8/group). (**I**) Lever presses and head entries did not differ between devalued (D) and non-devalued (ND) groups. FR = fixed ratio, VI = variable interval, RR = random ratio. Data are Means ±SEM.

The BALB/cJ strain took ∼13 sessions on average to attain criterion for Pavlovian discriminated approach, but with ∼20% of the mice tested being unable to reach the criterion even with prolonged training. Mice also passed through instrumental training within ∼13 sessions (Figure **4A**). On the PIT probe, there were significantly more AL presses during CS+ trials, than during CS− trials or during the ITI periods (main effect stimulus: F2,22 = 11.71, *p*<.01, followed by *post hoc* tests) (Figure **4B**). BALB/cJ mice pressed significantly more at the AL than the IL during CS+ (t = 14.67, df = 11, *p*<.01) and CS− (t = 4.83, df = 11, *p*<.01) trials and during ITI periods (t = 5.69, df = 11, *p*<.01). These mice also made significantly more HEs during presentation of the CS+ than during ITI periods, but not during CS− trials (main effect stimulus: F2,22 = 5.60, *p*<.05, followed by *post hoc* tests) (Figure **4C**).

#### Acquisition, extinction and reinstatement

BALB/cJ mice attained acquisition criterion in ∼11 sessions (Figure **4D**) and extinction of instrumental responding to criterion occurred within ∼7 sessions (Figure **4E**). BALB/cJ mice showed modest but significant reinstatement (5–7 responses over extinction) with all 3 procedures: Reward^6^+CR^6^ (t = 3.21, df = 8, *p*<.05), CR^30^ (t = 2.64, df = 9, *p*<.05) and Reward^6^+CR^30^ (t = 4.09, df = 8, *p*<.01).

#### Reinforcer devaluation

BALB/cJ mice monotonically increased lever pressing across instrumental sessions to a rate of ∼15 presses/min (Figure **4G**). Before the first devaluation pairing, both devalued and non-devalued groups ate most of the 50 pellets and the devalued group consumed slightly, but significantly, fewer pellets than the non-devalued group on the second pairing day (t = 3.71, df = 14, *p*<.01) (Figure **4H**). While the number of lever presses was again not different between the devalued -and non-devalued groups during the devaluation probe test in the conditioning chambers, devalued mice made significantly fewer HEs compared to non-devalued mice (t = 8.63, df = 14, *p*<.05) (Figure **4I**). Moreover, mice in the devalued group consumed significantly fewer pellets than those in the non-devalued condition during the probe for generalization to the conditioning chamber (t = 3.80, df = 14, *p*<.01), but not during the retention test in the home cages (Figure **4H**).

## Discussion

C57BL/6J is one of most commonly used inbred strains of mice, especially as a genetic background in mutant mouse lines [31], [32]. Confirming a few previous studies that have reported PIT in C57BL/6J and (mixed or congenic) C57BL/6J-background mutant mice using comparable procedures as employed here [e.g.], [ 33], [34]–[43], we found robust PIT in this strain. This was evidenced by increased instrumental response rates during presentations of an auditory cue previously associated with reward delivery, compared to presentations of a cue not associated with the reward. Furthermore, this cue-induced instrumental responding was specifically directed to the lever that had delivered reward during training although, interestingly, there was an increase in responding on the control lever during the no-CS periods; potentially reflecting a type of explorative response driven by the ‘release-from-stimulus-control’ during periods when no cue was available to predict either the availability or absence of reward. It is worth noting that, in addition to showing increased lever pressing during the CS+ (PIT), C57BL/6J also showed robust CS+-controlled Pavlovian approach or goal-tracking responses (head entries). The expression of PIT (instrumental responding) and Pavlovian approach have been reported to compete at the behavioral level [44]. Additionally, as PIT and CS-controlled goal-tracking rely on separate neurobiological mechanisms [45], observing both in the same mouse and during the same single test allows for powerful dissociations based on strain and/or genetic mutation [e.g.], [ 35,36]. Collectively, our PIT data demonstrate strong formation of associative representations underlying incentive motivation and robust expression of stimulus-controlled reward-seeking behaviors in the C57BL/6J strain.

This conclusion was bolstered by the performance of this strain in our instrumental stimulus-response paradigm where C57BL/6J mice readily learned to correctly respond to a visual stimulus on a touchscreen to obtain a food reward. In turn, when reinforcement was omitted, instrumental responding efficiently extinguished, indicating that a S-R association had been established during training. We have reported similar patterns in C57BL/6J and 129/SvImJ inbred mice [23] and C57BL/6J-background mutants [24], [25]. A novel observation here was that (extinguished) responding was robustly reinstated. Reinstatement was produced either by brief presentation of the US and the tone/light compound cue associated with the reward (conditioned reinforcer, CR), by brief US presentation followed by presentation of the CRs on all remaining trials, or by presentation of the CRs alone on all trials. Interestingly, the magnitude of responding during these different reinstatement conditions was not different in the C57BL/6J strain, indicating that exposure to the CRs alone was an effective re-energizer of responding, but that the continued presentation of the CRs after an initial exposure to the reward itself was not sufficient to further augment responding. The lack of any differences or additive effects could arguably reflect a behavioral ceiling effect, although this seems less likely as responding remained at around thirty percent of the maximum. Additional, controlled experimentation would be required to explore this issue further. It would also be useful in future experiments to include a CS− condition in order to confirm that CS-induced reinstatement of responding was explicitly due to its associative history with reward.

An interesting and somewhat surprising finding was that C57BL/6J mice were insensitive to outcome devaluation caused by repeated pairing of the food US with sickness induced by LiCl injection. This was indicated by the fact that neither instrumental responding (lever pressing) nor discriminated approach or goal-tracking behavior (magazine head-entries) were reduced in mice having undergone LiCl-food paired devaluation, relative to non-devalued control mice. It is important to emphasize, however, that the absence of any instrumental devaluation effect was not simply an artifact of a failure of mice to form an association between the food US and the experience of malaise, because mice in the devalued group clearly showed a persistent aversion to consuming the freely available reward in the home cage and in the operant chambers themselves. By definition then, these findings suggest that the instrumental reward-seeking response is, at least in part, habitual and driven by processes that are separate and divorced from the outcome.

On the other hand, the absence of a devaluation effect contrasts with previous reports of clear outcome devaluation induced using sensory-specific satiety, rather than LiCl pairings, in C57BL/6J-background mice [e.g.], [ 30], [39], [41], [43], [46,47]. In these studies, instrumental lever press responses were markedly reduced in mice that had been sated with the reward (but not a different natural reinforcer) prior to the probe test. Certain instrumental training variables, including the schedule of reinforcement [30], [48] and the overall number of reinforcers earned [28], are known to determine sensitivity to outcome devaluation. However, these factors cannot account for the discrepancy between previous studies because we controlled for reinforcers earned and used the same training procedure and random ratio schedule previously shown to be effective in producing satiety-induced devaluation in C57BL/6J-background mice [30]. Whatever the case, these contrasting findings indicate that differences exist between sensitivity to malaise- and satiety-induced devaluation that render the former ineffective in reducing instrumental lever press behavior in C57BL/6J mice.

This conclusion was not limited to C57BL/6J mice, as we found that DBA/2J mice also failed to show altered responding following reinforcer devaluation (again, despite clear evidence of the formation of a successful food-sickness pairing). To our knowledge, this is the first published report of a reinforcer devaluation procedure used with this mouse strain. DBA/2J mice have been widely used in behavioral neuroscience and, within addiction research, have been heavily studied because of an aversion (relative to C57BL/6J) to orally consumed alcohol [49]. This strain (along with C57BL/6J) also has an important role in behavior genetic studies as one of the parental strains of the BXD recombinant panels employed to identify sources of genetic variation underlying, for example, behavioral responses to abused drugs including alcohol [50], nicotine [51] and psychostimulants [52].

In addition to insensitivity to malaise-induced reward devaluation, we found that DBA/2J mice were similar to C57BL/6J mice in that they by and large showed robust PIT, extinction and reinstatement. The purpose of the current study was not to cross-compare strains as strains were not tested in a fully counterbalanced design, precluding direct statistical comparison. However, testing was done under identical conditions and informal visual comparison of the data suggests that DBA/2J mice were similar to C57BL/6J mice on the majority of behavioral measures. One exception was that DBA/2J mice seemed slower to extinguish the instrumental response – requiring more sessions to extinguish this response than to acquire it (C57BL/6J mice showed the opposite pattern). The DBA/2J strain also failed to show significant reinstatement of this response when exposed to conditioned reinforcers alone. While this suggests weak conditioned reinforcement in this paradigm, it cannot simply be explained by a more general failure to form cue-reward relationships, *per se*, as PIT was clearly intact in these mice. Additionally, although both rely broadly on intact corticostriatal circuitry function, numerous studies in rats and mice show that PIT and conditioned reinforcement are dissociable at the systems, receptor and molecular levels [5], [35], [36]. Thus, further studies will be needed to clarify whether the DBA/2J strain has a genuine deficit in certain forms of reinstatement.

The third strain we characterized on these tasks was BALB/cJ. BALB/cJ has been well-studied for its heightened anxiety-like behavior and stress reactivity in comparison to, for example, C57BL/6J [e.g.], [ 53], [54,55]. As such, this strain could prove valuable to studies aimed at identifying genetic and molecular factors modulating stress effects on cognitive and executive deficits in addiction [for further discussion see 56]. We found that BALB/cJ mice displayed good PIT, extinction and reinstatement. During the PIT probe, BALB/cJ mice did not engage in exploration of the inactive lever during the CS− and no-CS periods, which is reminiscent of their reduced tendency to explore in anxiety-provoking environments. The BALB/cJ strain also exhibited poor discriminated Pavlovian approach behavior during the PIT probe, i.e., these mice did not engage in significantly more reward-seeking during the CS+ than the CS−.

As with the other two strains, instrumental lever pressing was undiminished by reinforcer devaluation. Interestingly, however, there was a significant decrease in magazine entries in devalued BALB/cJ mice (although the strength of the LiCl-induced illness-food pairing seemed relatively weak in these mice and was not expressed on the long-term retention test). This indicates that at least one component of the behavioral repertoire of BALB/cJ mice in this test was sensitive to the current reward value. But, again, instrumental lever pressing and Pavlovian-approach behaviors are dissociable processes [57]–[59], and their differential sensitivity to outcome devaluation is not unprecedented. For example, Nelson and Killcross found that repeated amphetamine treatment rendered rats insensitive to either malaise- or satiety-induced devaluation of lever pressing but not head entry behavior [28]. As discussed by these authors, magazine entry behavior may be more resistant to changes in reward value either due to its proximity to the reward and/or because it has a Pavlovian approach component [see 60]. In this context, our data suggest that the BALB/cJ strain may provide an interesting genetic model to explore the nature of this dissociation in future studies.

In summary, the current study describes a set of paradigms for assaying various operant-based reward-related behaviors in three of the most commonly used inbred mouse strains. We describe a procedure for demonstrating PIT, and a method for studying acquisition, extinction and multiple forms of reinstatement of an instrumental touchscreen response. While we were unable to demonstrate malaise-induced devaluation of an instrumental response in any of our strains (although Pavlovian approach responses were sensitive to diminished outcome value in BALB/cJ mice) we found no reason to conclude that mice were unable to form the necessary food-malaise association and the negative results more likely point to more complex factors needing additional study. These procedures provide a useful platform for future studies using the mouse as a model species to elucidate the critical neural, molecular and genetic factors subserving reward-related behaviors, and ultimately provide new insights into maladaptive manifestations of motivated behaviors such as drug addiction.
